# Single-cell transcriptomics reveals cell atlas and identifies cycling tumor cells responsible for recurrence in ameloblastoma

**DOI:** 10.1038/s41368-024-00281-4

**Published:** 2024-02-29

**Authors:** Gan Xiong, Nan Xie, Min Nie, Rongsong Ling, Bokai Yun, Jiaxiang Xie, Linlin Ren, Yaqi Huang, Wenjin Wang, Chen Yi, Ming Zhang, Xiuyun Xu, Caihua Zhang, Bin Zou, Leitao Zhang, Xiqiang Liu, Hongzhang Huang, Demeng Chen, Wei Cao, Cheng Wang

**Affiliations:** 1 Hospital of Stomatology, Sun Yat-sen University, Guangzhou, China; 2 Guangdong Provincial Key Laboratory of Stomatology, Sun Yat-sen University, Guangzhou, China; 3 Guanghua School of Stomatology, Sun Yat-sen University, Guangzhou, China; 4 Department of Periodontics, Affiliated Stomatology Hospital of Guangzhou Medical University, Guangzhou Key Laboratory of Basic and Applied Research of Oral Regenerative Medicine, Guangzhou, China; 5 Institute for Advanced Study, Shenzhen University, Shenzhen, China; 6 Center for Translational Medicine, The First Affiliated Hospital, Sun Yat-sen University, Guangzhou, China; 7 State Key Laboratory of Ophthalmology, Zhongshan Ophthalmic Center, Sun Yat-sen University, Guangzhou, China; 8 Department of Oral and Maxillofacial Surgery, Nanfang Hospital, Southern Medical University, Guangzhou, China; 9 Department of Oral and Maxillofacial & Head and Neck Oncology, Shanghai Ninth People’s Hospital, Shanghai Jiao Tong University School of Medicine, Shanghai, China; 10 National Center for Stomatology, National Clinical Research Center for Oral diseases, Shanghai Key Laboratory of Stomatology, Shanghai, China

**Keywords:** Cancer, Medical research

## Abstract

Ameloblastoma is a benign tumor characterized by locally invasive phenotypes, leading to facial bone destruction and a high recurrence rate. However, the mechanisms governing tumor initiation and recurrence are poorly understood. Here, we uncovered cellular landscapes and mechanisms that underlie tumor recurrence in ameloblastoma at single-cell resolution. Our results revealed that ameloblastoma exhibits five tumor subpopulations varying with respect to immune response (IR), bone remodeling (BR), tooth development (TD), epithelial development (ED), and cell cycle (CC) signatures. Of note, we found that CC ameloblastoma cells were endowed with stemness and contributed to tumor recurrence, which was dominated by the EZH2-mediated program. Targeting EZH2 effectively eliminated CC ameloblastoma cells and inhibited tumor growth in ameloblastoma patient-derived organoids. These data described the tumor subpopulation and clarified the identity, function, and regulatory mechanism of CC ameloblastoma cells, providing a potential therapeutic target for ameloblastoma.

## Introduction

Ameloblastoma (AM) is the most common odontogenic epithelial tumor and is characterized by severe local bone destruction and a high recurrence rate. It is thought to arise from remnants of odontogenic epithelium and usually occurs in jaw bones, leading to severe facial deformity and dysfunction.^1–3^ The treatment for AM mainly relies on surgery, but the recurrence rate for conventional AM is almost 38%, even when radical surgery is performed.^4–6^ Moreover, tumor resection always results in loss of facial bones and causes severe facial deformity and dysfunction. Therefore, there is an unmet need to develop a new treatment modality for AM to improve clinical outcomes. Interestingly, BRAF inhibitors offer promising results in the treatment of BRAF-positive AM, indicating the feasibility of treating AM by targeting tumor driver genes.^7–10^ These findings emphasize the need for a better understanding of mechanisms governing the progression of AM, which may provide effective therapeutic strategies for the eradication of AM.

Single-cell transcriptomics has shown promising value in exploring intratumoral heterogeneity (ITH) and cellular crosstalk within the tumor microenvironment (TME) in many cancer types, including head and neck tumors.^11–14^ Increasing scRNA-seq studies of human tumors have revealed new insights into tumor composition, cancer stem cells, and identified cell populations that drive invasion, metastasis, drug resistance, and tumor plasticity. However, the genetic and functional heterogeneity at single-cell resolution have never been explored in AM. Here, we performed scRNA-seq to better understand ITH and the TME of AM at single-cell resolution. Our data reveal the transcriptomic profiles of the multicellular ecosystem of AM, distinguish diverse tumor, stromal, and immune cells. In total, nine major cell clusters were identified, and the cellular properties of tumor cells were characterized. Tumor cells varied in their expression of immune response (IR), bone remodeling (BR), tooth development (TD), cell cycle (CC), and epithelial development (ED) programs. Of note, the subset of CC AM cells was identified and confirmed to be highly tumorigenic. They are governed by an EZH2-mediated regulatory circuit and correlate with aggressive behavior and local recurrence of AM. Inhibition of EZH2 significantly suppresses the proliferation and tumorigenic capability of AM, suggesting the potential application of EZH2 inhibitors for AM treatment. Taken together, our results are the first to uncover the cellular and molecular characteristics of AM at single-cell resolution, which provides deeper insight into the heterogeneity and diversity of AM and may be helpful for therapeutic method development.

## Results

### Single-cell transcriptomic profiling of the multicellular ecosystem of ameloblastoma

**Fig. 1:**
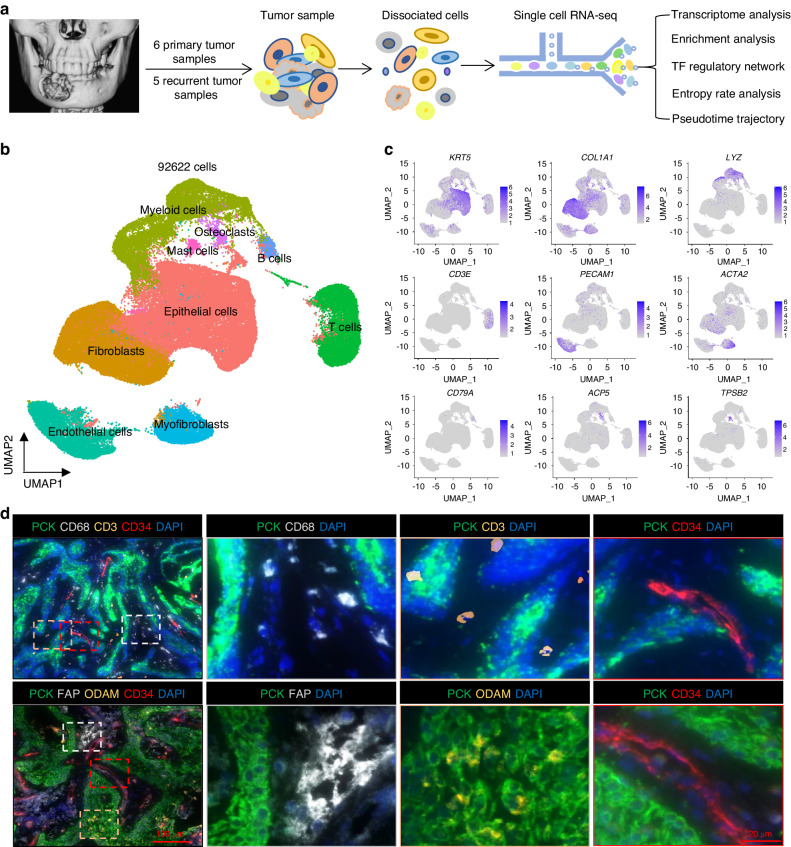
Single-cell transcriptomic profiling of the multicellular ecosystem of ameloblastoma. **a** Workflow showing the collection and processing of fresh patient samples from 11 tumors for scRNA-seq. (**b**) UMAP plot of 92,622 single cells from scRNA-seq labeled by cell type with different colors. **c** UMAP plot showing the expression of selected marker genes in the 9 clusters. (**d**) Representative images of multiplex immunohistochemistry showing the expression of PCK, CD3, CD34, CD68, ODAM, FAP in ameloblastoma; scale bar, 100 μm, and 20 μm

To investigate the cellular diversity of AM, we generated scRNA-seq profiles for tumors from 11 patients (Table S1) using the 10× Genomics Chromium platform (Fig. 1a). After quality filtering (Fig. S1A) and doublets removal, a total of 92,622 single cells from the AM were profiled. Overall, nine major cell types were identified according to graph-based clustering and dimensionally reduction with UMAP and then further annotated based on the expression of canonical gene markers and assessment using the SingleR package.^15^ (Fig. 1b, c, Fig. S1B–J). We identified epithelial cells expressing *KRT5, KRT6A, KRT8, KRT13, KRT14, KRT15, KRT16, KRT18*, and *KRT19*, fibroblasts expressing *COL1A1, COL1A2, LUM*, and *DCN*, lymphoid cells represented by T cells expressing *CD3D, CD3E, CD3G* and *CD2*, a myeloid population expressing *LYZ, HLA-DRA, HLA-DPA1*, and *HLA-DQA1*, mast cells expressing *TPSAB1, TPSB2*, and *MS4A2*, B cells expressing *CD79A, MS4A1*, and *CD37*, endothelial cells expressing *VWF, PECAM1, CDH5*, and *ENG*, and myofibroblasts expressing *ATCT2, MYL9*, and *MCAM, RGS5*. Notably, osteoclasts expressing *ACP5, CA2, MMP9*, and *SPP1* were also identified, highlighting the activity of bone resorption. Obviously, each of the 9 clusters contained cells from different patients, indicating that cell types and expression states in the tumor microenvironment are largely consistent across AM and do not represent patient-specific subpopulations or batch effects (Fig. S2A–C). However, the proportions of these cells varied in different patients. Multiplex immunohistochemistry (IHC) staining further identified the distribution of the main cell types, including PCK for epithelial cells, CD3 for T cells, CD34 for endothelial cells, CD68 for myeloid cells, FAP for fibroblasts (Fig. 1d). In addition, odontogenic ameloblast-associated protein ODAM were also stained to confirm the odontogenic origin of tumors (Fig. 1d). Next, we extracted all epithelial cells based on initial clustering and cell type identification and performed inferred large-scale chromosomal copy number variation (CNV) analysis. As expected, epithelial cells harbored many more CNVs in chromosomes than nonepithelial cells (Fig. S2D). Of note, the inferred CNV data analysis revealed copy number amplification on chromosomes 12 and 17, but significant deletions along chromosomes 6 and 10 were observed. Similar genomic alterations were also observed in AM by using various methods from previous studies.^16–19^ Taken together, these results provide a representative cellular landscape for AM, which comprises epithelial cells (tumor cells), fibroblasts, myofibroblasts, myeloid cells, lymphoid cells, mast cells, B cells, and osteoclasts.

### Single-cell transcriptomic analysis identifies intratumoral heterogeneity and diversity in ameloblastoma

**Fig. 2:**
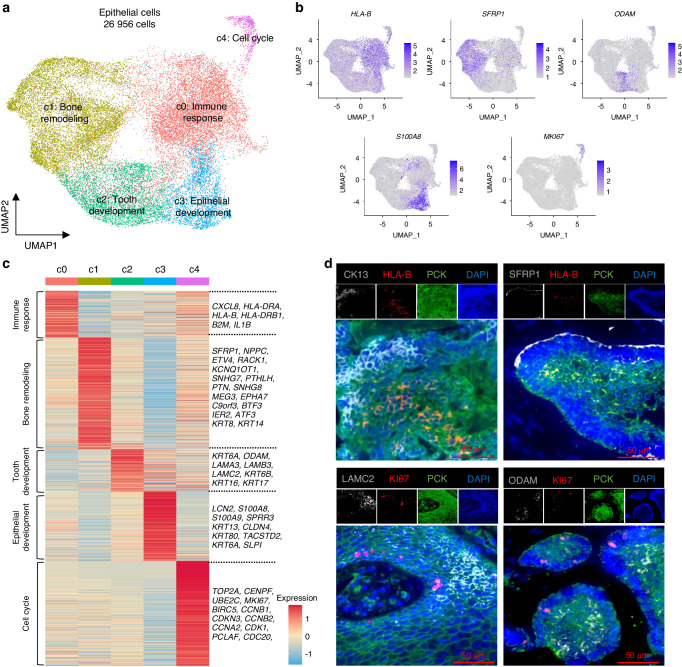
Single-cell transcriptomic analysis identifies intratumoral heterogeneity and diversity in ameloblastoma. **a** UMAP plot classifying 26,956 epithelial cells into five clusters with immune response (IR), bone remodeling (BR), tooth development (TD), epithelial development (ED), and cell cycle (CC). **b** UMAP plot displaying the expression of selected marker genes in different epithelial cell types. **c** Heatmap showing the differently expressed genes of each cluster, representative marker genes in the right panel. **d** Representative images of multiplex immunohistochemistry showing the expression of PCK, HLA-B, KRT13 (CK13), SFRP1, ODAM, LAMC2, and KI67 in ameloblastoma; scale bar, 50 μm

After re-clustering epithelial cells, 5 major epithelial subclusters were observed (Fig. 2a–c). Cells in c0 expressed high levels of *CXCL8*, *HLA-DRA, HLA-B*, *HLA-C, B2M,* and *IL1B*, indicating an epithelial cell with immune features, which was also observed in nasopharyngeal carcinoma.^20^ and our previous study on murine head and neck squamous cell carcinoma.^14^ Gene ontology (GO) analysis and gene set variation analysis (GSVA) analysis showed that the immune response was mainly correlated with this subset of cells (Fig. S3A, B), implying their potential function in modulating the tumor immune microenvironment of AM. Cells in c1 displayed high expression of genes related to bone remodeling and suppression of osteogenesis (*SFRP1*, *NPPC*, *RACK1*, SNHG7, *PTHLH,* and *PTN*), which represents the epithelial cell type with osteogenic inhibitory potentials.^21,22^ GO analysis and GSVA showed that bone remodeling-associated signatures and cell polarity-related pathways were enriched in c1 cells, indicating the interaction between c1 cells and bone remodeling (Fig. S3A, B). Cells in c2 expressed high levels of *KRT6A*, *ODAM, LAMA3, LAMB3,* and *LAMC2*, representing an ameloblast-like epithelial cell signature^23,24^ with partial epithelial-mesenchymal transition (pEMT) characteristics^11,25^ that might be involved in cell-cell adhesion and tooth mineralization and implying cytoskeletal organization and cell migration (Fig. S3A, B). Cells in c3 expressed *S100A8, SPRR3, S100A9, KRT13*, and *S100A7*, which are markers for differentiated epithelial cells involved in epithelial junctions (Fig. S3A, B). Cells in *c4* expressed high levels of *TOP2A, CENPF, UBE2C, MKI67, BIRC5, CCNB1, CDKN3,* and *CCNB2* representing a high-cycling cell type significantly enriched for cell division and cell cycle pathways (Fig. S3A, B). Then, we defined the c0-c4 cell cluster as immune response (IR), bone remodeling (BR), tooth development (TD), epithelial development (ED), and cell cycle (CC) according to the expression of well-known marker genes, GO analysis, and GSVA described above mentioned. Tumor cell clustering was also driven predominantly by cell type, but not batch effects (Fig. S3C). To validate these findings, multiplex IHC staining was then performed to detect the 5 tumor clusters by using marker genes. As shown in Fig. 2d, all five clusters of tumor epithelial cells were indeed observed in AM samples by using marker genes (HLA-B for IR, SFRP1 for BR, KRT13 for ED, ODAM for TD, LAMC2 for TD, and KI67 for CC). Although the 5 tumor clusters displayed notable transcriptomic characteristics, there were some transcriptional features shared by tumor clusters belonging to different cellular states, indicating that transformation of cellular states might occur among clusters. Then, we performed the signaling entropy rate analysis and showed that the cell cycle tumor cluster had the highest differentiation potency (Fig. S3D), suggesting that the CC AM cells represented the starting state during AM development. Then, Monocle3 was used to depict the cellular state changes of tumor cells, which showed that tumor cells started from CC AM cells and evolved toward BR states (Fig. S3E). These findings suggest that AM displays high intratumoral heterogeneity and diversity.

### The CC subpopulation is correlated with recurrence and endowed with stemness in ameloblastoma

**Fig. 3:**
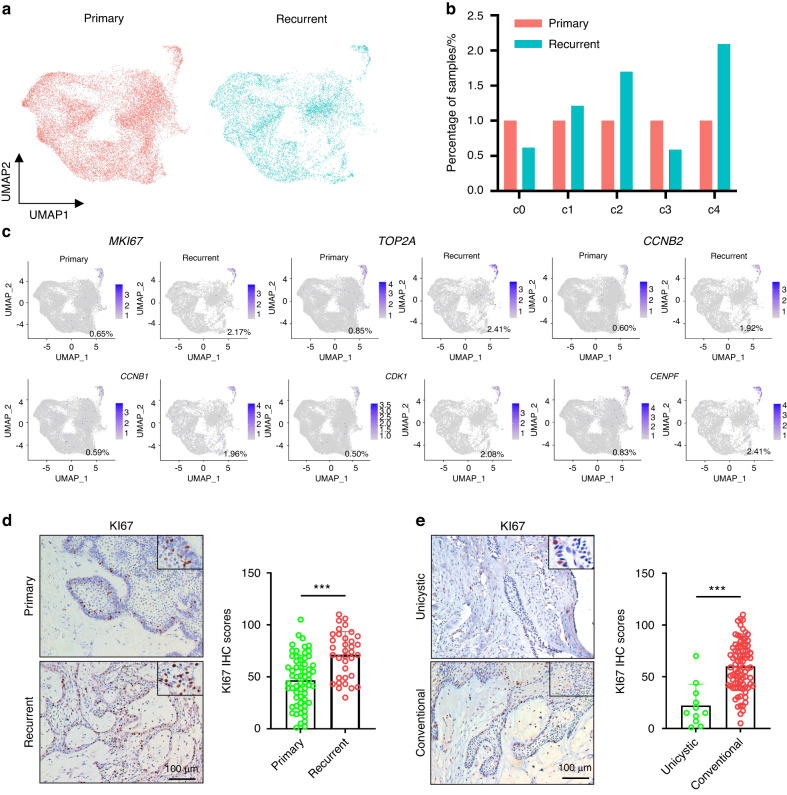
The CC subpopulation is correlated with recurrence in ameloblastoma. **a** UMAP plot showing all epithelial cells that were divided into primary and recurrent samples. **b** Bar plot showing the relative percent of different tumor cell subpopulations in primary and recurrent samples. **c** UMAP plot showing the expression of *MKI67, TOP2A, CCNB2, CCNB1, CDK1*, and *CENPF* in epithelial cells derived from primary and recurrent samples. **d** Representative images of KI67 IHC staining in primary and recurrent ameloblastoma, scale bar 100 μm (left panel). Quantification of KI67 expression in primary and recurrent ameloblastoma (right panel). ***P < 0.001 by Student’s *t* test. **e** Representative images of KI67 IHC staining in unicystic and conventional ameloblastoma; scale bar, 100 μm (left panel). Quantification of KI67 expression in unicystic and conventional ameloblastoma (right panel), ***P < 0.001 by Student’s *t* test

**Fig. 4:**
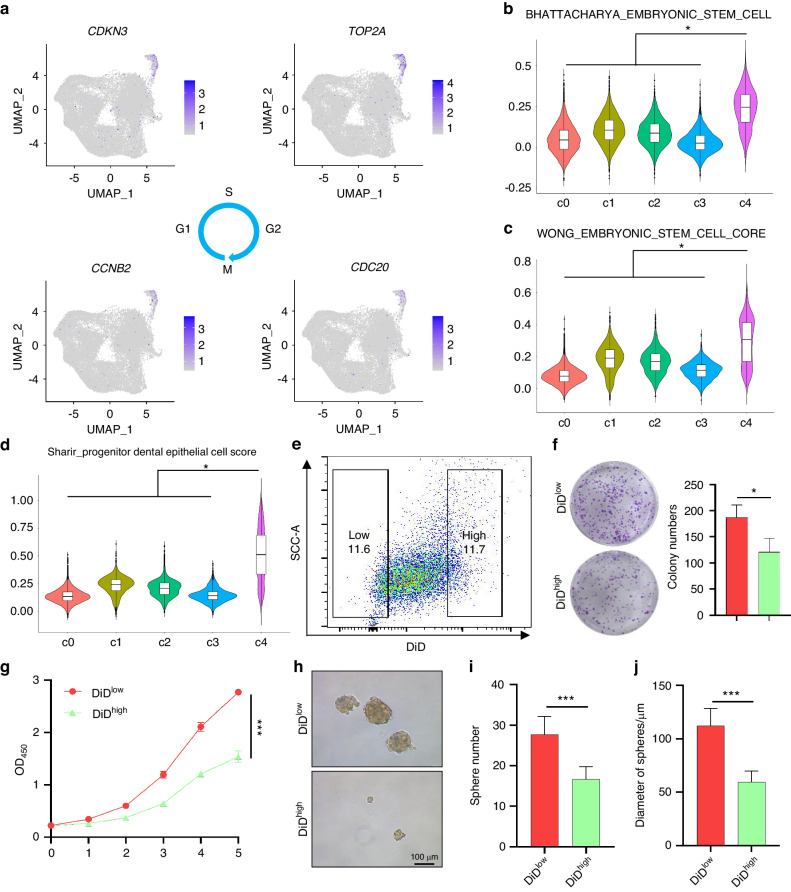
CC ameloblastoma cells is endowed with stemness in ameloblastoma. **a** UMAP plot showing the expression of selected genes related to the cell cycle in CC AM cells. **b**–**d** Violin plot of stemness-related gene signature scores in the five epithelial clusters, *P < 2.22e-16 by pairwise Wilcoxon rank-sum tests. **e** DiD^low^ and DiD^high^ AM cells sorted by using DiD staining. **f** Representative images and quantification of colony formation in sorted DiD^low^ and DiD^high^ AM cells, *P < 0.05 by Student’s *t* test. **g** The CCK8 assay showing the proliferative activity of sorted DiD^low^ and DiD^high^ AM cells, ***P < 0.001 by two-way ANOVA. **h** Representative images showing spheres from sorted DiD^low^ and DiD^high^ AM cells; scale bar, 100 μm. **i** Quantification of sphere numbers from sorted DiD^low^ and DiD^high^ AM cells, ***P < 0.001 by Student’s *t* test. **j** Quantification of sphere diameter from sorted DiD^low^ and DiD^high^ AM cells, ***P < 0.001 by Student’s *t* test

**Fig. 5:**
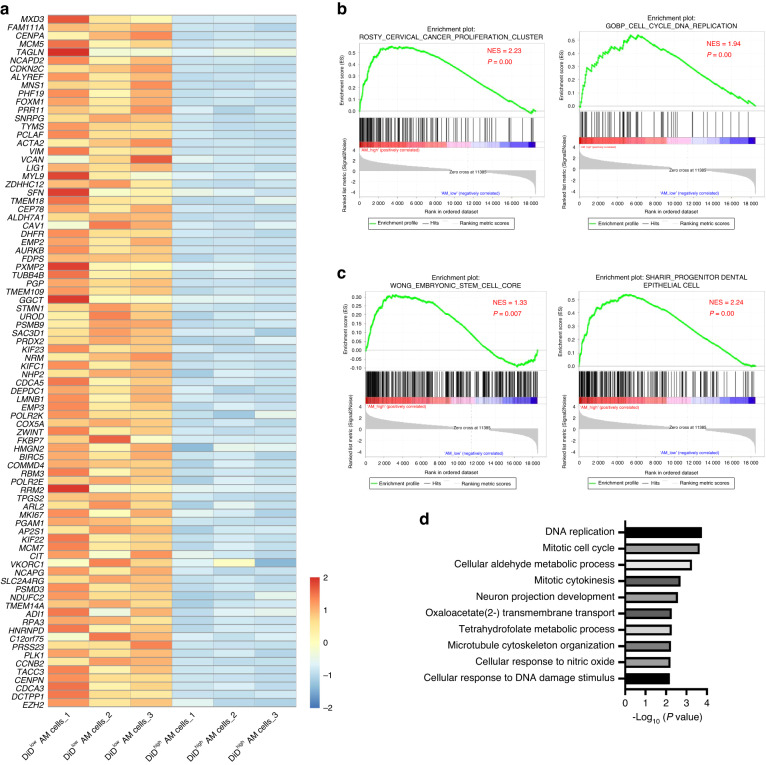
Molecular characteristics of the sorted DiD^low^ ameloblastoma cells. **a** Heatmap showing the expression of selected genes in sorted DiD^low^ and DiD^high^ AM cells from RNA-seq. **b** GSEA showing that the cancer proliferation signature and cell cycle-associated signature were significantly enriched in sorted DiD^low^ AM cells compared with sorted DiD^high^ AM cells. **c** GSEA showing that the stemness gene signature was significantly enriched in sorted DiD^low^ AM cells compared to sorted DiD^high^ AM cells. **d** Bar plots showing the top 10 biological process from GO analysis for DiD^low^ AM cells. The highly expressed genes in sorted DiD^low^ AM cells used for GO analysis (log_2_fold change >1, *P* value < 0.05)

It is well-known that AM is a locally aggressive odontogenic tumor with a high risk of recurrence, which is a major challenge for clinical treatment. We further investigated the distribution of tumor cell clusters in primary (*n* = 6) and recurrent (*n* = 5) AM (Table S1). As shown in Fig. 3a, b, the c4 cluster (CC AM cells) was dramatically increased, but the c3 cluster (ED cells) was decreased in recurrent AM compared to primary samples. As expected, *MKI6*7^+^, *TOP2A*^*+*^, *CCNB2*^+^, *CCNB1*^*+*^, *CDK1*^*+*^, and *CENPF*^*+*^ AM cells were also increased in recurrent AM compared with primary samples (Fig. 3c). These findings indicate that CC AM cells are responsible for tumor recurrence. Then, KI67 (marker gene for CC AM cells) immunohistochemical staining was performed to detect CC AM cells in 96 AM patient samples (Table S2). As shown in Fig. 3d, KI67-positive tumor cells were significantly increased in recurrent AM compared to primary AM. Similar results were also observed in conventional AM when compared with unicystic AM (Fig. 3e), providing a rational explanation for the higher rate of recurrence in conventional AM than unicystic AM. Interestingly, we also found that successive phases of the cell cycle loop were formed within the cluster of CC AM cells, suggesting that some cells at the end of the cell cycle return to their original state, reflecting self-renewal (Fig. 4a).^26^ We identified S, G2/M and M/G1 phases by the expression of *CDKN3* (G1 and S), *TOP2A* (end of S phase to G2), *CDC20* (end of G2 phase to M entry) and *CCNB2* (end of M phase to G1 entry). Notably, CC AM cells exhibited high expression of the embryonic stem cell gene signature.^27,28^ (Fig. 4b, c) and dental epithelial stem cell gene signature^26^ (Fig. 4d). These findings indicated that CC AM cells might house tumor-initiating cells in AM, which is consistent with dental epithelial stem cells in a mouse model^26^ and reinforced by our signaling entropy rate analysis and pseudotime trajectories analysis (Fig. S3D, E). To validate these findings, CC AM cells were sorted by FACS based on DiD staining and then assessed by using sphere formation, colony formation and CCK8 assays. We found that sorted DiD^low^ AM cells (CC AM cells with high cell cycling) did harbor many more tumor-initiating cells with high tumorigenicity and high proliferative activity than DiD^high^ AM cells (non-CC AM cells with low cell cycling) (Fig. 4e–j). To further characterize sorted CC AM cells, we compared the gene expression profiles of sorted CC AM cells and non-CC AM cells by RNA-seq. As shown in Fig. 5a, genes upregulated in sorted CC AM cells were mainly associated with proliferation and the cell cycle. Importantly, gene set enrichment analysis (GSEA) also revealed that cell cycle-associated signatures (Fig. 5b) and stemness gene signatures (Fig. 5c) were significantly enriched in sorted CC AM cells. GO analysis showed that upregulated genes in sorted CC AM cells were enriched with DNA replication pathway, mitotic cell cycle pathway and mitotic cytokines pathway (Fig. 5d), which is consistent with the characteristics of c4 cell clusters based on our scRNA-seq data. To investigate the function of CC AM cells in AM patients, we downloaded the gene expression matrix of GSE132472 from gene expression omnibus (GEO) database.^29^ After GSVA analysis by using CC AM cells signature, the patients were splitting into two groups (high CC score group and low CC score group) (Fig. S4A). Consistently, GO analysis displayed that the highly expressed genes in high CC score group were enriched in negative regulation of apoptotic process and positive regulation of cell proliferation pathway (Fig. S4B). GSEA also showed that cell cycle-associated signature and stemness gene signature were enriched in high CC score group (Fig. S4C). Collectively, these data identify a CC population of AM cells endowed with stemness and high proliferative activity that contributes to tumor recurrence.

### Targeting EZH2 inhibits proliferation and stemness in ameloblastoma cells

**Fig. 6:**
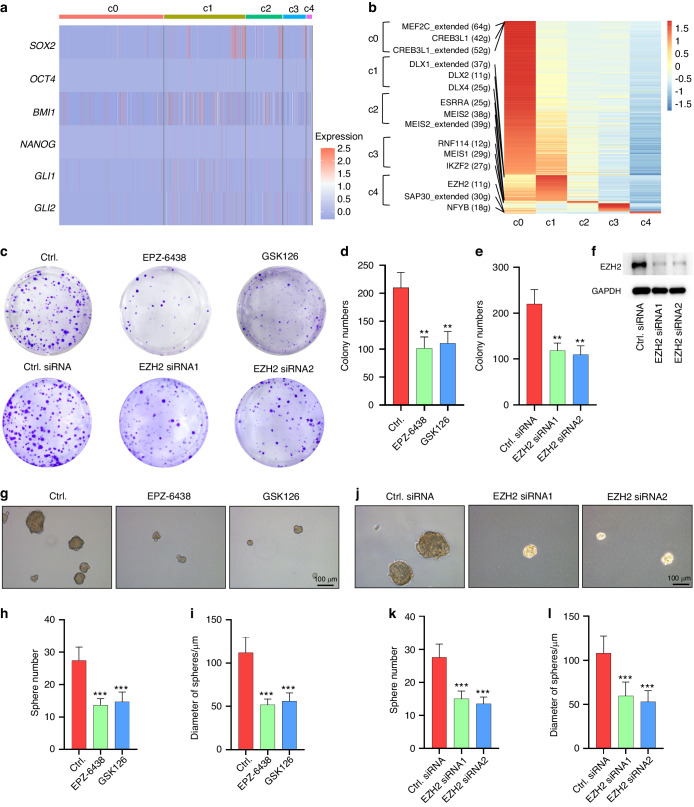
Targeting EZH2 inhibits proliferation and stemness in ameloblastoma cells. **a** Heatmap of the expression of stem cell-associated transcription factors in tumor cells. **b** Heatmap of the differentiated regulons of each epithelial cluster inferred by SCENIC. **c** Representative images of colony formation of sorted DiD^low^ AM cells treated with EZH2 siRNAs or EZH2 inhibitors. **d**, **e** Quantification of colony numbers of sorted DiD^low^ AM cells treated with EZH2 siRNAs or EZH2 inhibitors, **P < 0.01 by one-way ANOVA. **f** Western blot images showing EZH2 expression of DiD^low^ AM cells treated with EZH2 siRNAs. **g** Representative images showing spheres of sorted DiD^low^ AM cells treated with two EZH2 inhibitors; scale bar, 100 μm. **h**, **i** Quantification of sphere number and diameter in sorted DiD^low^ AM cells treated with or without EZH2 inhibitors, ***P < 0.001 by one-way ANOVA. **j** Representative images showing spheres of sorted DiD^low^ AM cells treated with two different EZH2 siRNAs; scale bar, 100 μm. (**k, l**) Quantification of sphere number and diameter in sorted DiD^low^ AM cells transfected with siRNA control or EZH2 siRNAs, ***P < 0.001 by one-way ANOVA

Next, the key regulators of CC AM cells were further investigated. Similar to a previous study of cycling dental epithelial stem cells,^26^ CC AM cells lacked expression of classic stem cell-associated transcription factors, including *SOX2*, *BMI1*, *GLI1*, *NANOG,* and *OCT4* (Fig. 6a). Therefore, we performed single-cell regulatory network inference and clustering (SCENIC), which identified EZH2 as the most significant TF potentially controlling CC AM cells (Fig. 6b). The effect of EZH2 on proliferation and stemness was then examined by pharmacological inhibition and genetic silencing of EZH2. As shown in Fig. 6c–l, inhibition of EZH2 with two different pharmacological inhibitors or siRNAs significantly suppressed cell proliferation and sphere formation in sorted CC AM cells. As expected, the RNA-seq results showed that knockdown of EZH2 significantly inhibited genes associated with proliferation and cell cycling (Fig. S5A). Notably, GSEA showed that cell proliferation or cycling gene signatures (Fig. S5B) and stemness gene signatures (Fig. S5C) were also suppressed in AM cells treated with EZH2 siRNAs compared to the control siRNAs. GO analysis showed that cell division and pathway and mitotic spindle organization pathway are functionally inhibited by targeting EZH2 with specific siRNAs (Fig. S5D), which is also consistent with the characteristics of c4 cell clusters of AM and CC AM cells sorted by FACS. Taken together, these findings imply that CC AM cells exhibit the properties of tumor-initiating cells and high proliferative activity, which can be suppressed by targeting EZH2.

### EZH2 is a novel therapeutic target for ameloblastoma

**Fig. 7:**
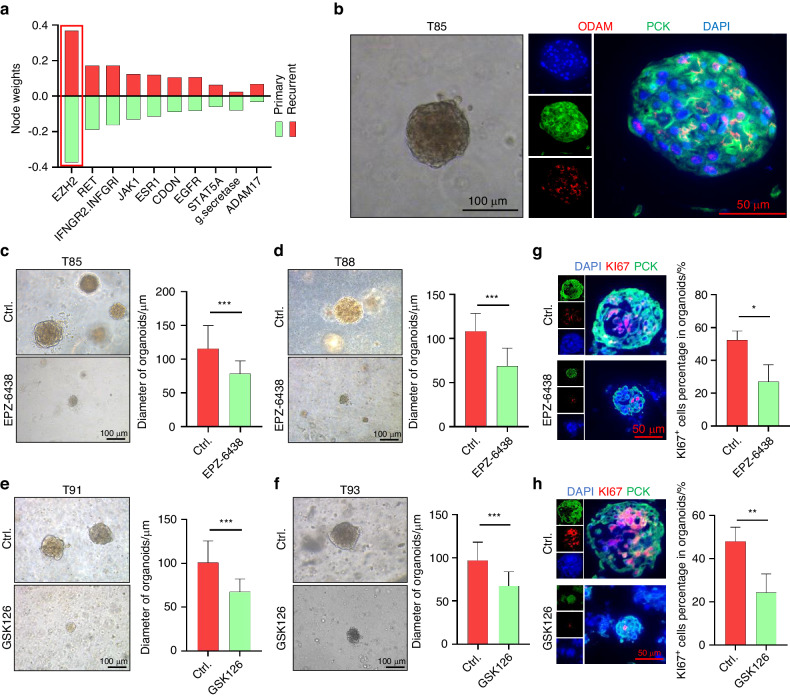
EZH2 is a novel therapeutic target for ameloblastoma. **a** KPNN analysis showing top 10 nodes with the most differential weights between primary and recurrent samples. **b** Representative images of organoids derived from T85 ameloblastoma samples; scale bar, 100 μm (left panel). Immunofluorescent staining showing the expression of PCK and ODAM in ameloblastoma organoids; scale bar, 50 μm (right panel). **c**, **d** Representative images and quantification of T85 and T88 ameloblastoma organoids treated with or without EPZ-6438, scale bar, 100 μm, ***P < 0.001 by Student’s *t* test. **e**, **f** Representative images and quantification of T91 and T93 ameloblastoma organoids treated with or without GSK126; scale bar, 100 μm, *** *P* < 0.001 by Student’s *t* test. **g**, **h** Immunofluorescent staining assay showed KI67 and PCK expression in organoids; scale bar, 50 μm (left panel). The percentage of KI67 expression in organoids with or without EZH2 inhibitors, *P < 0.05 and **P < 0.01 by Student’s *t* test (right panel)

**Fig. 8:**
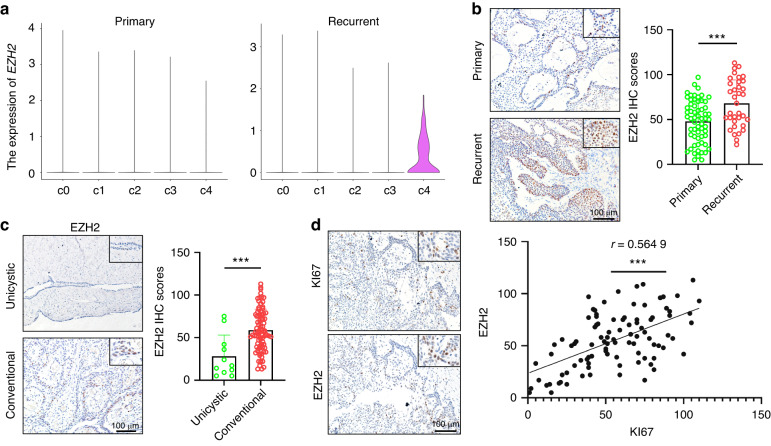
EZH2 expression is increased in recurrent AM and positively correlated with KI67 expression. **a** Violin plot showing the expression of EZH2 in the five clusters after splitting epithelial cells into primary and recurrent groups. **b** Representative images of EZH2 IHC staining in primary and recurrent ameloblastoma; scale bar, 100 μm (left panel). Quantification of EZH2 IHC staining in primary and recurrent ameloblastoma, ***P < 0.001 by Student’s *t* test (right panel). **c** Representative images of EZH2 IHC staining in unicystic and conventional ameloblastoma; scale bar, 100 μm (left panel). Quantification of EZH2 IHC staining in unicystic and conventional ameloblastoma, ***P < 0.001 by Student’s *t* test (right panel). **d** Representative images showing the same region of KI67 and EZH2 IHC staining in serial sections; scale bar, 100 μm (left panel). The correlation analysis between the expression of KI67 and EZH2 was assessed, ***P < 0.001 by Pearson (right panel)

Our findings show that CC AM cells characterized by high proliferative activity and stemness are dominated by EZH2, which indicates that EZH2 may serve as an effective therapeutic target for AM. To further investigate the hub genes contributing to the recurrence of AM, we performed a deep learning analysis based on our scRNA-seq datasets.^30^ Strikingly, EZH2 was again identified as one of the most critical driver genes for the recurrence of AM (Fig. 7a), which strengthens our hypothesis that EZH2 may be an effective therapeutic target for AM. To test this hypothesis, AM patient-derived organoids (APDOs) (Fig. 7b and Fig. S6A–C) were cultured, validated, and treated with EZH2 inhibitors. As shown in Fig. 7c–f, pharmacological inhibition of EZH2 significantly impaired the formation and growth of organoids in APDOs. Immunofluorescent staining also showed that KI67 expression was suppressed by pharmacological inhibition of EZH2 in AM PDOs (Fig. 7g, h). Importantly, our scRNA-seq analysis also showed that EZH2 expression was dramatically increased in recurrent AM compared to primary AM (Fig. 8a). Consistent with these findings, the expression of EZH2 was increased in recurrent AM tissue compared to primary AM tissues (Fig. 8b). Similar results were also observed in conventional AM when compared with unicystic AM (Fig. 8c). As expected, EZH2 predominantly colocalized with KI67 in both APDOs and AM samples (Fig. S6D, E). Importantly, the expression of EZH2 was positively correlated with KI67 expression in AM patients (Fig. 8d).

## Discussion

AM is a benign but locally aggressive and destructive epithelial odontogenic tumor that usually starts in jaw bones, often near or including teeth. Although this neoplasm was first described in 1827 by Cusack,^31,32^ the landscapes of cell constituents and their genetic heterogeneity are still mysterious and have not been clarified. Here, we performed scRNA-seq to construct a single-cell transcriptomic atlas of cell subpopulations within AM. In this study, we identified nine major clusters of cells with UMAP clustering that contribute to AM lesions and further analyzed the tumor cells and their tumor microenvironment (TME). The cellular atlas of tumor cells revealed intratumor heterogeneity and diversity, which may provide therapeutic targets for AM in the future.

Among our key findings is the identification of the heterogeneity and diversity of tumor epithelial cells within AM. Our data revealed five subsets of tumor cells characterized by different transcriptomic states, including IR, BR, TD, ED and CC AM cells. Notably, a subset of high-cycling AM cells (CC AM cells), but not slow-cycling AM cells, was identified to maintain active self-renewal and tumor-initiating potentials, which showed high tumorigenic capability and was correlated with recurrence of AM. Strikingly, classic stemness-driven transcription factors are lacking in CC AM cells, including *SOX2*, *BMI1*, *GLI1*, *OCT4* and *NANOG*. Of note, *SOX2, BMI1*, and *GLI1* have been considered to be stem cell markers for dental epithelial cells in previous studies,^33–37^ but are not consistent with an updated study based on scRNA-seq, which confirmed that actively cycling dental epithelial cells are stem cells with the capacity of self-renewal that can differentiate into both functional ameloblasts and the surrounding non-ameloblast epithelium.^26^ EZH2 was further identified to be the key transcription factor driving cycling of AM cells based on our unbiased scRNA-deq data, which was also nominated as one of the critical hub genes for recurrence by using machine learning. EZH2 is an enzymatic catalytic subunit of polycomb repressive complex 2 (PRC2) that can regulate downstream target gene expression by trimethylation of Lys-27 in histone 3 (H3K27me3), which serves pivotal roles in cell proliferation, self-renewal and invasion.^38–41^ We found that EZH2 was predominantly expressed in CC AM cells and that targeting EZH2 inhibited cell proliferation, sphere formation and organoid formation. As expected, the expression of EZH2 was positively correlated with KI67 expression and increased in recurrent AM compared to primary AM. These findings indicate that CC AM cells harbor tumor-initiating cells that are responsible for AM recurrence and that targeting EZH2 suppresses the stemness of AM cells, suggesting that inhibition of EZH2 is a promising approach for AM treatment.

In summary, our work first provides an atlas of tumor cells at single-cell resolution in AM. Of note, a CC AM cell subset with properties of tumor-initiating cells controlled by EZH2 is confirmed to be responsible for tumor recurrence, suggesting that targeting CC AM cells and EZH2 are promising approaches for AM treatment.

### Limitations of the study

Although we reveal that CC AM cells are endowed with stemness and contribute to tumor recurrence in AM, we are unable to observe the cell fate of CC AM cells by lineage tracing in vivo due to a lack of mouse models for AM. In addition, AM harbors different oncogenic mutations, including *BRAF*, *SMO*, *RAS*, and *FGFR2* mutations.^7,8,42^ AM with different mutation statuses might have distinct features, which need to be investigated in the future.

## Materials and methods

### Human ameloblastoma samples

Human ameloblastoma samples were collected from Guanghua School of Stomatology, Sun Yat-sen University, and protected by using MACS Tissue Storage Solution (Miltenyi, Cat#130-100-008). All fresh surgical tissues were diagnosed with ameloblastoma by two pathologists. For scRNA-seq analysis, eleven tumor samples were prepared for scRNA-seq (Table S1). This study was approved by the Medical Ethics Committee of Hospital of Stomatology Sun Yat-Sen University.

### Cell lines

The ameloblastoma immortalized cell line hTERT^+^-AM^43^ was cultured in DMEM (Thermo Fisher Scientific, Cat#C11995500BT) with 10% FBS (Thermo Fisher Scientific, Cat#A3160801) and 1% Penicillin/Streptomycin (Thermo Fisher Scientific, Cat#15140-122). All cells were cultured at 37 °C in a cultivation cabinet with 5% CO_2_.

### Preparation of single-cell suspensions

The fresh tissues were washed with phosphate-buffered saline (PBS; BOSTER, Cat#AR0030-1), cut into small pieces (<1 mm^3^) and dissociated using a Tumor Dissociation Kit (Miltenyi, Cat#130-095-929) according to the manufacturer’s instructions. After tissue dissociation, the cell suspension was filtered using a 40-µm nylon mesh (BD Falcon, Cat#352350). Dissociated cells were pelleted and washed with cold DMEM, after which the filtered cells were resuspended with ACK Lysing Buffer (Thermo Fisher Scientific, Cat#A1049201) for 5 minutes to remove red blood cells. Cell viability was confirmed to be >85% in all samples by using Trypan blue staining. Finally, the single-cell suspension was used for scRNA-seq droplet capture. During the period of tissue dissociation, the cells were always kept on ice, and the processing time was less than 2 hours.

### Single-cell RNA sequencing

The single-cell suspension was processed by 10× Genomics Chromium Single-Cell 3′ reagents Kit version 3 (10× Genomics, Pleasanton, CA, USA) according to the manufacturer’s protocol. Cells were loaded onto a chromium single-cell chip with a target number of 6 000–12 000 cells. The libraries were sequenced on the Illumina Nova6000 system with 150-bp paired-end sequencing. Raw reads were processed with Cell Ranger (version 3.1.0 for T35 data, version 3.0.1 for T45 data, version 4.0.0 for T51, T56, T58 and T68 data, version 6.1.1 for T107, T109 and T110, version 7.1.0 for T115 and T116 data) and mapped to the human genome reference sequence (GRCH38) to generate a file containing a gene expression matrix, a gene table, and a barcode table.

### ScRNA-seq data processing

The Seurat R package (version 4.0.0) was used to analyze the gene-barcode expression matrix of each sample in R software (version 4.0.2). Cells with <200 or >9 000 expressed genes were eliminated from downstream analysis. Moreover, cells with >10% mitochondrial genes mapped or >7% hemoglobin genes were also filtered out. The ribosomal and mitochondrial genes were deleted in the following step. Then, the potential doublets in individual samples were identified and removed by using the DoubletFinder package (version 2.0.3) with default parameters.^44^ After the strict quality control above, a total of 92,622 remaining cells were used for the following analysis. The number of genes (nFeature), number of UMI (nCount), percent of mitochondrial (percent.mt), and percent of hemoglobin (percent.HB) of each cell in each sample was showed by violin plot (Fig. 1a).

### Data integration and cell type identification

All samples were processed with normalization, variance stabilization by using sctransform (version 0.3.2) and merged with the IntegrateData function into one Seurat object. For dimensionality reduction, principal component analysis (PCA) was used to summarize the top 3 000 highly variable genes (HVGs), and then the ElbowPlot function was used to estimate each principal component. A total of 20 principal components were selected for uniform manifold approximation and projection (UMAP). The cell clusters were identified by using the FindClusters function with a resolution of 1.0 after computing shared nearest neighbors based on the Louvain algorithm. To assign cell type, the FindAllmarkers function was used to find the differentially expressed genes (DEGs) in Seurat with default parameters. We then annotated the clusters by using the well-known marker genes reported in the literature. We identified tumor cells expressing *KRT5, KRT8, KRT14, KRT15* and *KRT19*; fibroblasts expressing *COL1A1, COL1A2*, *DCN* and *LUM*; T cells expressing *CD2*, *CD3D, CD3E* and *CD3G*; endothelial cells expressing *CDH5*, *PECAM1*, *VWF*, and *ENG*; myeloid cells expressing *CD74*, *LYZ* and MHC class II genes (including *HLA-DRA*, *HLA-DPA1* and *HLA-DQA1*); myofibroblasts expressing *ACTA2*, *MYL9*, *MCAM* and *RGS5*; osteoclasts expressing *CA2*, *ACP5*, *SPP1*, and *MMP9*; mast cells expressing *TPSB2*, *MS4A2*, and *TPSAB1;* and B cells expressing *CD79A*, *CD37*, and *MS4A1*.

To further dissect the major cell types, the cells belonging to each cell type were extracted with an “RNA” assay. Then, the cells from each sample were processed with sctransform and merged as described above. The subclusters were determined by repeating dimensionality reduction and unsupervised re-clustering. For cell types, subclusters were annotated to cell subtypes according to the top differentially expressed genes and biological functions. During the aforementioned re-clustering for epithelial cells, we also observed a small cluster, which was inferred to be doublets and removed for further analysis as described above.^14,45^

### Single-cell copy number variation (CNV) analysis

Initial CNVs for each epithelial cell were estimated with the inferCNV package (version 1.6.0) of R with default parameters as described previously.^11,46^ The CNV scores of myeloid cells and T cells were calculated as a CNV control.

### GSVA analysis

The relative pathways of each epithelial cluster were performed by GSVA R package (version 1.44.2) with the non-parametric and unsupervised algorithm. The signature gene sets of biological processes was derived from MSigDB gene ontology (GO) datasets.^47^

### Signal entropy rate analysis

The SCENT R package (version 1.0.3) was performed to estimate the differentiation potency of each cluster.^48^ The signal entropy rate of single cells was calculated with default parameters.

### SCENIC analysis

SCENIC analysis was performed as described above.^49^ We used the SCENIC R package (version 1.2.1) to calculate the AUCell scores of each identified regulon with recommended parameters. The heatmap was used to display the differentiated regulons of each cluster.

### Trajectory analysis

Trajectory analysis was performed by using monocle3 R package (version 1.3.1) for epithelial cells.^50^ In the trajectory analysis, the batch effect was removed by running the align_cds() function. The analysis results, including pseudotime score and trajectory were merged into the UMAP graph. The CC AM cells were selected as the starting cells by running order_cells() function. The plot_cells() function was applied to visualize the cells.

### Organoid culture

The AM tissues were shredded and incubated at 37 °C with Collagenase Type IV for 50 minutes (Stemcell, Cat#07909). After digestion was completed, 10 mL DMEM/F12 (Thermo Fisher Scientific, Cat#C11330500BT) was added to dilute the collagenase. The suspension was strained through a 100 µm sieve (Falcon, Cat#352360) and centrifuged at 1 000 r/min for 5 minutes. The pellet was resuspended in BioCoat MATRIGEL MATRIX (Corning, Cat#354234) mixed with organoid medium (1:1), and then the miscible liquid was plated on 24-well culture plates and placed in an incubator at 37 °C for 30 minutes. Organoids were cultured in self-configured medium. The medium contained DMEM/F12 (Thermo Fisher Scientific, Cat#C11330500BT), 1× B27 supplement (Thermo Fisher Scientific, Cat#12587010), 1.25 mmol/L N-acetyl-L-cysteine (Sigma, Cat#A7250), 10 mmol/L nicotinamide (Sigma, Cat#N0636), 50 ng/mL human EGF (PeproTech, Cat#AF-100-15), 500 nmol/L A83-01 (PeproTech, Cat#9094360), 10 ng/mL human FGF10 (PeproTech, Cat#100-26-5), 5 ng/mL human FGF2 (Sino Biological Inc, Cat#10014-HNAE), 1 μmol/L prostaglandin E2 (MCE, Cat#HY-101952), 0.3 μmol/L CHIR 99021 (Sigma, Cat#SML1046), 1 μmol/L forskolin (Abcam, Cat#ab120058), 50 ng/mL R-spondin (R&D Systems, Cat#3266-RS) and 25 ng/mL Noggin (PeproTech, Cat#120-10 C). To aid outgrowth of organoids, 10 μmol/L Rho-associated kinase (ROCK) inhibitor Y-27632 (TargetMol, Cat#T1725) was added to the medium after the first week.^51,52^ The medium was changed every 2–3 days, and the organoids were passaged every 1–2 weeks.

### Tumorsphere formation assay

For the tumorsphere formation assay, cells were seeded into ultralow attachment plates and cultured in serum-free DMEM/F12 (Thermo Fisher Scientific, Cat#C11330500BT) supplemented with 1% B27 supplement (Thermo Fisher Scientific, Cat#12587010), 1% penicillin/streptomycin (Thermo Fisher Scientific, Cat#15140-122), human recombinant epidermal growth factor (20 ng/mL; PeproTech, Cat#PHG0311) and human recombinant basic fibroblast growth factor (20 ng/mL; PeproTech, Cat#100-18B-10) in a cultivation cabinet with 5% CO_2_ at 37 °C. The number and diameter of spheres were evaluated after 10 days.

### Cell proliferation assay

For the cell proliferation assay, 1 000 cells were seeded in each well of a 96-well plate and cultured in a cultivation cabinet. The proliferation ability was assessed by using CCK8 reagent (Dojindo, Cat#CCK8-500). After every 24 hours, 10 μL reaction agent mixed with 90 μL DMEM was added to each well and incubated for 2 hours at 37 °C. A microplate reader (Infinite M200 PRO) was used to measure the optical density at 450 nm.

### Colony formation assay

For colony formation, 2 mL of cell medium containing 1 000 cells was seeded in 6-well plates and cultivated for ~10 days. Then, the cells were fixed by using 4% paraformaldehyde (Biosharp, Cat#BL539A) for 30 minutes and stained with 0.1% crystal violet (Roles-Bio, Cat#RBG1019-100 mL). The colony numbers of each well were counted by using ImageJ.

### Immunohistochemistry and H&E staining

The ameloblastoma samples were obtained from Guanghua School of Stomatology, Sun Yat-sen University. The tumors were fixed in 4% paraformaldehyde for 24 h, dehydrated, embedded in paraffin, and sectioned at 4 μm thickness. After dewaxing, the sections were subjected to heat-induced epitope recovery (Zsbio, Cat#ZLI-9065) and incubated in 3% H_2_O_2_ for 10 minutes. Then, the sections were washed and incubated with primary antibodies against KI67 (Novus, Cat#NB500-170; 1:200) and EZH2 (Cell Signaling Technology, Cat#5246 S; 1:200) overnight at 4 °C. After incubation with the secondary biotinylated antibody (Zsbio, Cat#PV-6001-18) for 40 minutes, horseradish peroxidase conjugation was carried out with a DAB kit (Zsbio, Cat#ZLI-9017). The scores of each sample were assessed via the staining intensity and area of tumor cells as described previously.^25^ For the H&E assay, paraffin-embedded tumor sections were stained with an H&E kit (Solarbio, Cat#G1120-100) following the manufacturer’s instructions.

### Multiplex immunohistochemistry (IHC) staining

The ameloblastoma paraffin sections were stained using a multi-labeled immunohistochemistry staining kit (Akoya, Cat#NEL811001KT) according to the manufacturer’s protocols. Briefly, the sections were incubated in 3% H_2_O_2_ for 10 minutes for the first time. Every time the sections were incubated with primary antibodies, they were first subjected to heat-induced epitope recovery and blocked with 5% BSA. The HRP conjugate and three wavelengths (520, 570, and 650 nm) were then utilized to attach the different primary antibodies, which included PANCK (Proteintech, Cat#26411-1-AP; 1:1 000), CD3 (Proteintech, Cat#17617-1-AP; 1:800), CD34 (Abcam, Cat#ab81289; 1:800), CD68 (Abcam, Cat#ab213363; 1:800), FAP (Cell Signaling Technology, Cat#E1V9V; 1:800), ODAM (Affinity, Cat#DF13204; 1:800), KI67 (Novus, Cat#NB500-170; 1:800), SFRP1 (Abcam, Cat#ab126613; 1:800), HLA-B (Thermo Fisher Scientific, Cat#PA5-35345; 1:800), LAMC2 (Abcam, Cat#ab210959; 1:800), EZH2 (Cell Signaling Technology, Cat#5246 S; 1:800), and KRT13 (Proteintech, Cat#10164-2-AP; 1:800). Finally, the cell nuclei were stained with DAPI (Solarbio, Cat#C0065-50ml).

### Immunostaining

The tumorsphere immunostaining assay was performed as described previously.^53^ All primary antibodies, including PANCK (Santa Cruz, Cat#sc-8018) and ODAM (Affinity Biosciences, Cat#DF13204-100ul), were used at dilutions of 1:200.

### Flow cytometry and cell sorting

hTERT^+^-AM cells were stained with DiD dye (Thermo Fisher, Cat#V22887), according to the manufacturer’s directions. The DiD dye was lost as cells divided, which allowed highly proliferating cells to be identified and distinguished from slowly dividing cells.^54–56^ Briefly, 1 mL serum-free DMEM containing 1×10^6^ cells was incubated with DiD dye (1:200) at 37 °C for 10 minutes. After washing three times, the cells were plated in the sterile cell culture dishes and passaged every 2 days. After 10 days, the DiD^high^ AM cells and DiD^low^ AM cells were sorted by a FACSVantage instrument (BD FACSAriaFusion) and used for the functional experiment.

### siRNA transfection

AM cells were cultured in DMEM with 10% FBS as described above. For EZH2 siRNA transfection, 10 nM siRNAs were transfected with Lipofectamine^TM^ RNAiMAX Transfection Reagent (Thermo Fisher Scientific, Cat#13778150) according to the manufacturer’s protocols. The sequences of EZH2 siRNAs were 5′-CCAACACAAGUCAUCCCAUUATT-3′ and 5′-CCCAACAUAGAUGGACCAAAUTT-3′. The negative control was purchased from Sangon Biotech, and the sequence was 5′-UUCUCCGAACGUGUCACGUTT-3′.

### Western blot

For western blotting, the proteins of AM were extracted using RIPA lysis buffer (Beyotime, Cat#P0013B). After separation by SDS–PAGE, the proteins were transferred to PVDF membranes. Then, the membranes were blocked with 5% milk at room temperature and incubated with primary antibodies overnight at 4 °C, followed by incubation with secondary antibodies for 1 hour. The following primary antibodies were used: EZH2 (Cell Signaling Technology, Cat#5246 S; 1:2 000) and GAPDH (Proteintech, 10494-1-AP).

### RNA-seq analysis

For RNA-seq analysis, total RNA was isolated using TRIzol reagent (Thermo Fisher Scientific, Cat#15596018) according to the manufacturer’s protocols. The RNA was then qualified and quantified by using a NanoDrop and Agilent 2100 bioanalyzer (Thermo Fisher Scientific, MA, USA). RNA library preparation was performed by using the MGIseq-500 platform (MGI Technology, MGISEQ-500). The gene count matrix was acquired by comparison with GRCH38. The DEGs of RNA-seq were obtained by using the DESeq2 (version 1.28.1) package in R.

### AI analysis

For artificial intelligence (AI) analysis, the Knowledge-primed neural networks (KPNN) analysis was processed as described before.^30^ Briefly, the expression matrix of ameloblastoma tumor cells of primary and recurrent samples was extracted. The node weights were then obtained by using the KPNN Python package (https://github.com/epigen/KPNN). The top 10 nodes with the most differential weights between primary and recurrent samples were used to show the results.

### Statistical analyses

Data are shown as the mean ± SD. All statistical analyses were performed using R and GraphPad Prism 9 for Windows (GraphPad software, Inc.). In Figure Legends, the results of statistical analyses are explained. Several gene lists for the stem-like module were obtained from Wong’s study,^28^ Bhattacharya’s research^27^, and Sharir’s work.^26^ The gene oncology (GO) analysis was performed by Database for Annotation, Visualization and Integrated Discovery (DAVID) website (https://david.ncifcrf.gov/).

### Supplementary information

Table S1

Table S2

Table S3

Supplementary Figures

## Data Availability

scRNA-seq and RNA-seq data were deposited at the National Genomics Data Center (NGDC) under access numbers HRA004732 and HRA004835.

## Materials availability

The reagents and R package used in this study are described in Table S3 and are available upon request. All requests must execute a suitable Materials Transfer Agreement. Further information and requests for reagents may be directed to and will be fulfilled by the corresponding author, Prof. Cheng Wang (wangch75@mail.sysu.edu.cn).
