# Supplementary material for: Single-cell transcriptomics reveals cell atlas and identifies cycling tumor cells responsible for recurrence in ameloblastoma
Source: Int J Oral Sci. 2024 Feb 29;16:21. doi: 10.1038/s41368-024-00281-4 (PMC10904398; doi:10.1038/s41368-024-00281-4)
Supplement: Supplementary file 1 — Table S1 [file 41368_2024_281_MOESM1_ESM.docx]

Table S1. Characteristics of 11 patients with ameloblastoma for single-cell RNA sequencing.

| patient # | gender | age | classification | location | primary/recurrent |
| --- | --- | --- | --- | --- | --- |
| T35 | female | 43y | conventional | mandible | primary |
| T45 | male | 15y | conventional | mandible | recurrent |
| T51 | female | 46y | conventional | mandible | recurrent |
| T56 | female | 30y | conventional | mandible | recurrent |
| T58 | female | 33y | conventional | mandible | primary |
| T68 | male | 48y | conventional | mandible | primary |
| T107 | female | 27y | conventional | mandible | recurrent |
| T109 | male | 68y | conventional | mandible | primary |
| T110 | male | 36y | conventional | maxilla | recurrent |
| T115 | male | 51y | conventional | mandible | primary |
| T116 | female | 57y | conventional | mandible | primary |
