# Supplementary material for: Single-cell transcriptomics reveals cell atlas and identifies cycling tumor cells responsible for recurrence in ameloblastoma
Source: Int J Oral Sci. 2024 Feb 29;16:21. doi: 10.1038/s41368-024-00281-4 (PMC10904398; doi:10.1038/s41368-024-00281-4)
Supplement: Supplementary file 2 — Table S2 [file 41368_2024_281_MOESM2_ESM.docx]

Table S2. Characteristics of 96 patients with ameloblastoma for immunohistochemical analysis

| patient # | gender | age | classification | location | primary/recurrent |
| --- | --- | --- | --- | --- | --- |
| A1 | male | 30y | unicystic | mandible | primary |
| A2 | male | 19y | unicystic | mandible | primary |
| A3 | male | 27y | unicystic | mandible | primary |
| A4 | male | 27y | unicystic | mandible | primary |
| A5 | female | 61y | unicystic | mandible | primary |
| A6 | female | 25y | unicystic | mandible | primary |
| A7 | male | 25y | unicystic | mandible | primary |
| A8 | male | 33y | unicystic | mandible | primary |
| A9 | male | 46y | unicystic | mandible | primary |
| A10 | female | 21y | unicystic | mandible | primary |
| A11 | female | 41y | unicystic | mandible | primary |
| A12 | male | 14y | conventional | mandible | primary |
| A13 | male | 25y | conventional | maxilla | primary |
| A14 | male | 25y | conventional | mandible | primary |
| A15 | male | 30y | conventional | mandible | primary |
| A16 | male | 52y | conventional | mandible | primary |
| A17 | female | 28y | conventional | mandible | primary |
| A18 | male | 25y | conventional | mandible | primary |
| A19 | female | 11y | conventional | mandible | primary |
| A20 | male | 40y | conventional | maxilla | primary |
| A21 | male | 47y | conventional | mandible | primary |
| A22 | female | 18y | conventional | mandible | primary |
| A23 | female | 30y | conventional | mandible | primary |
| A24 | male | 25y | conventional | mandible | primary |
| A25 | male | 35y | conventional | mandible | primary |
| A26 | male | 31y | conventional | mandible | primary |
| A27 | male | 19y | conventional | mandible | primary |
| A28 | male | 28y | conventional | mandible | primary |
| A29 | male | 36y | conventional | mandible | primary |
| A30 | female | 24y | conventional | mandible | primary |
| A31 | male | 18y | conventional | mandible | primary |
| A32 | female | 25y | conventional | mandible | primary |
| A33 | female | 43y | conventional | mandible | primary |
| A34 | male | 28y | conventional | mandible | primary |
| A35 | male | 13y | conventional | mandible | primary |
| A36 | male | 32y | conventional | mandible | primary |
| A37 | male | 29y | conventional | mandible | primary |
| A38 | male | 20y | conventional | mandible | primary |
| A39 | male | 27y | conventional | mandible | primary |
| A40 | male | 23y | conventional | mandible | primary |
| A41 | male | 18y | conventional | mandible | primary |
| A42 | male | 26y | conventional | mandible | primary |
| A43 | male | 29y | conventional | mandible | primary |
| A44 | male | 21y | conventional | mandible | primary |
| A45 | male | 12y | conventional | mandible | primary |
| A46 | male | 55y | conventional | mandible | primary |
| A47 | male | 19y | conventional | mandible | primary |
| A48 | male | 39y | conventional | mandible | primary |
| A49 | female | 60y | conventional | mandible | primary |
| A50 | female | 11y | conventional | mandible | primary |
| A51 | female | 27y | conventional | mandible | primary |
| A52 | female | 33y | conventional | mandible | primary |
| A53 | female | 48y | conventional | mandible | primary |
| A54 | female | 60y | conventional | mandible | primary |
| A55 | male | 45y | conventional | mandible | primary |
| A56 | female | 34y | conventional | mandible | primary |
| A57 | male | 53y | conventional | mandible | primary |
| A58 | male | 50y | conventional | mandible | primary |
| A59 | female | 41y | conventional | mandible | primary |
| A60 | female | 24y | conventional | mandible | primary |
| A61 | female | 49y | conventional | maxilla | primary |
| A62 | male | 32y | conventional | mandible | recurrent |
| A63 | female | 40y | conventional | mandible | recurrent |
| A64 | female | 65y | conventional | mandible | recurrent |
| A65 | female | 21y | conventional | mandible | recurrent |
| A66 | male | 26y | conventional | mandible | recurrent |
| A67 | male | 45y | conventional | mandible | recurrent |
| A68 | male | 20y | conventional | mandible | recurrent |
| A69 | male | 39y | conventional | mandible | recurrent |
| A70 | male | 35y | conventional | mandible | recurrent |
| A71 | female | 36y | conventional | mandible | recurrent |
| A72 | male | 54y | conventional | mandible | recurrent |
| A73 | male | 37y | conventional | mandible | recurrent |
| A74 | female | 50y | conventional | mandible | recurrent |
| A75 | female | 51y | conventional | mandible | recurrent |
| A76 | male | 40y | conventional | mandible | recurrent |
| A77 | female | 27y | conventional | mandible | recurrent |
| A78 | male | 67y | conventional | mandible | recurrent |
| A79 | female | 23y | conventional | mandible | recurrent |
| A80 | male | 57y | conventional | mandible | recurrent |
| A81 | male | 42y | conventional | mandible | recurrent |
| A82 | female | 64y | conventional | mandible | recurrent |
| A83 | female | 38y | conventional | mandible | recurrent |
| A84 | male | 13y | conventional | mandible | recurrent |
| A85 | male | 41y | conventional | mandible | recurrent |
| A86 | male | 21y | conventional | mandible | recurrent |
| A87 | male | 22y | conventional | mandible | recurrent |
| A88 | female | 28y | conventional | mandible | recurrent |
| A89 | female | 46y | conventional | mandible | recurrent |
| A90 | male | 35y | conventional | mandible | recurrent |
| A91 | male | 24y | conventional | mandible | recurrent |
| A92 | female | 59y | conventional | mandible | recurrent |
| A93 | female | 25y | conventional | mandible | recurrent |
| A94 | female | 16y | conventional | mandible | recurrent |
| A95 | female | 22y | conventional | mandible | recurrent |
| A96 | male | 34y | conventional | mandible | recurrent |
