# Supplementary material for: Single-cell transcriptomics reveals cell atlas and identifies cycling tumor cells responsible for recurrence in ameloblastoma
Source: Int J Oral Sci. 2024 Feb 29;16:21. doi: 10.1038/s41368-024-00281-4 (PMC10904398; doi:10.1038/s41368-024-00281-4)
Supplement: Supplementary file 3 — Table S3 [file 41368_2024_281_MOESM3_ESM.docx]

Table S3. Key resource and reagents

| REAGENT or RESOURCE | SOURCE | IDENTIFIER |
| --- | --- | --- |
| Antibodies | | |
| Rabbit polyclonal anti-CD3 | Proteintech | Cat#17617-1-AP; RRID: AB1939430 |
| Rabbit monoclonal anti-CD34 | Abcam | Cat#ab81289; RRID: AB_1640331 |
| Rabbit monoclonal anti-CD68 | Abcam | Cat#ab213363; RRID: AB_2801637 |
| Rabbit monoclonal anti-FAP | Cell Signaling  Technology | Cat#E1V9V; RRID: [AB_2904193](http://antibodyregistry.org/AB_10694683) |
| Rabbit polyclonal anti-SFRP1 | Abcam | Cat#ab126613; RRID: AB_11128257 |
| Rabbit polyclonal anti-Cytokeratin 13 | Proteintech | Cat#10164-2-AP; RRID: [AB_2134679](http://antibodyregistry.org/AB_2281020) |
| Mouse monoclonal anti-LAMC2 | Abcam | Cat#ab210959; RRID: [AB_2895141](http://antibodyregistry.org/AB_2895141) |
| Rabbit monoclonal anti-EZH2 | Cell Signaling  Technology | Cat#5246S; RRID: [AB_10694683](http://antibodyregistry.org/AB_10694683) |
| Rabbit polyclonal anti-KI67 | Novus | Cat#NB500-170; RRID: [AB_10001977](http://antibodyregistry.org/AB_10001977) |
| Rabbit polyclonal anti-ODAM | Affinity Biosciences | Cat#DF13204; RRID: [AB_2846164](http://antibodyregistry.org/AB_2846164) |
| Rabbit polyclonal anti-pan Cytokeratin | Proteintech | Cat#26411-1-AP; RRID: [AB_2880505](http://antibodyregistry.org/AB_2880505) |
| Rabbit polyclonal anti-HLA-B | Thermo Fisher Scientific | [Cat#PA5-35345; RRID: AB_2552655](http://antibodyregistry.org/AB_2552655) |
| Rabbit monoclonal anti-pan Cytokeratin | Santa Cruz Biotechnology | Cat#sc-8018; RRID: AB_627396 |
| Rabbit polyclonal antibody GAPDH | Proteintech | Cat#10494-1-AP; RRID: [AB_2263076](http://antibodyregistry.org/AB_2263076) |
| Biological Samples | | |
| Human Ameloblastoma primary samples | Guanghua School of Stomatology, Sun Yat-sen University | <https://www.zdkqyy.com/> |
| Chemicals, Peptides, and Recombinant Proteins | | |
| DMEM | Thermo Fisher Scientific | Cat#C11995500BT |
| DMEM/F-12 | Thermo Fisher Scientific | Cat#C11330500BT |
| Fetal Bovine Serum | Thermo Fisher Scientific | Cat#A3160801 |
| Penicillin–Streptomycin | Thermo Fisher Scientific | Cat#15140122 |
| EPZ-6438 | Selleck | Cat#S7128-10mg |
| GSK126 | Selleck | Cat#S7061-5mg |
| Trypsin-EDTA | BasalMedia | Cat#S310KJ |
| B-27 Supplement (50X), minus vitamin A | Thermo Fisher Scientific | Cat#12587010 |
| N-acetyl-L-cysteine | Sigma | Cat#A7250 |
| Nicotinamide | Sigma | Cat#N0636 |
| Recombinant Human EGF protein | PeproTech | Cat#AF-100-15 |
| A83-01 | PeproTech | Cat#9094360 |
| FGF10 | PeproTech | Cat#100-26-5 |
| Recombinant Human FGF2 protein | Sino Biological | Cat#10014-HNAE |
| Prostaglandin E2 | MCE | Cat#HY-101952 |
| CHIR 99021 | Sigma | Cat#SML1046 |
| Forskolin | Abcam | Cat#ab120058 |
| Recombinant Human R-spondin protein | R&D Systems | Cat#3266-RS |
| Recombinant Human Noggin | PeproTech | Cat#120-10C |
| Y-27632 | TargetMol | Cat#T1725 |
| DiD | Thermo Fisher Scientific | Cat#V22887 |
| Tissue-TEK OCT Compound | SAKURA | Cat#4583 |
| Tissue Storage Solution | Miltenyi Biotech | Cat#130-100-008 |
| 4% Paraformaldehyde | Biosharp | Cat#BL539A |
| Collagenase Type IV | Stemcell | Cat#07909 |
| ACK Lysing Buffer | Thermo Fisher Scientific | Cat#A1049201 |
| BioCoat MATRIGEL MATRIX | Corning | Cat#354234 |
| Critical Commercial Assays | | |
| OPAL 7-COLOR MANUAL IHC KITakp | Akoya | Cat#NEL811001KT |
| Human Tumor Cell Dissociation Kit | Miltenyi Biotech | Cat#130-095-929 |
| Chromium Next GEM Single Cell 3ʹ GEM, Library & Gel Bead Kit v3.1 | 10X Genomics | Cat#PN-1000121 |
| Deposited Data | | |
| Raw data files for scRNA sequencing | This paper | [HRA004732](https://ngdc.cncb.ac.cn/gsub/submit/bioproject/PRJCA017335) |
| Raw data files for RNA-seq of high-cycling hTERT^+^-AM cells transfected with control siRNA or EZH2 siRNA, and raw data files for RNA-seq of high and low-cycling hTERT^+^-AM cells | This paper | HRA004835 |
| Experimental Models: Cell Lines | | |
| hTERT^+^-AM | Guanghua School of Stomatology, Sun Yat-sen University | N/A |
| Oligonucleotides | | |
| EZH2 siRNA-1  5’-CCAACACAAGUCAUCCCAUUATT-3’ | Sangon Biotech | N/A |
| EZH2 siRNA-2  5’-CCCAACAUAGAUGGACCAAAUTT-3’ | Sangon Biotech | N/A |
| Software and Algorithms | | |
| Cell Ranger versions 3.1.0/3.0.1/4.0.0/6.11/7.1.0 | 10X Genomics | <https://www.10xgenomics.com/> |
| Seurat versions 4.0.0 | Seurat R package | <https://satijalab.org/seurat/>；  RRID:SCR_016341 |
| DoubletFinder version 2.0.3 | DoubletFinder R package | <https://github.com/chris-mcginnis-ucsf/DoubletFinder> |
| Infercnv version 1.6.0 | Infercnv R package | <https://github.com/broadinstitute/infercnv>; RRID:SCR_021140 |
| GSVA version 1.44.2 | GSVA R package | <https://github.com/rcastelo/GSVA>; |
| SCENT version 1.0.3 | SCENT R package | https://github.com/aet21/SCENT |
| SCENIC version 1.2.1 | SCENIC R package | <https://github.com/aertslab/SCENIC/>；  RRID:SCR_017247 |
| Monocle3 version 1.3.1 | Monocle3 R package | <http://cole-trapnell-lab.github.io/monocle-release/monocle3/> |
| FlowJo | FlowJo | <https://www.flowjo.com/> |
| Graphpad Prism 9 software | GraphPad Software,  Inc. | <https://www.graphpad.com/scientific/software/prism/> |
| SingleR version 1.4.1 | SingleR R package | <https://github.com/dviraran/SingleR> |
| DESeq2 version 1.28.1. | DESeq2 R package | <https://github.com/mikelove/DESeq2> |
| GSEA software | Broad Institute | <https://www.gsea-msigdb.org/gsea/index.jsp> |
