# Supplementary material for: Single-cell transcriptomics reveals cell atlas and identifies cycling tumor cells responsible for recurrence in ameloblastoma
Source: Int J Oral Sci. 2024 Feb 29;16:21. doi: 10.1038/s41368-024-00281-4 (PMC10904398; doi:10.1038/s41368-024-00281-4)
Supplement: Supplementary file 4 — Supplementary Figures [file 41368_2024_281_MOESM4_ESM.pdf]

## Supplementary Figures

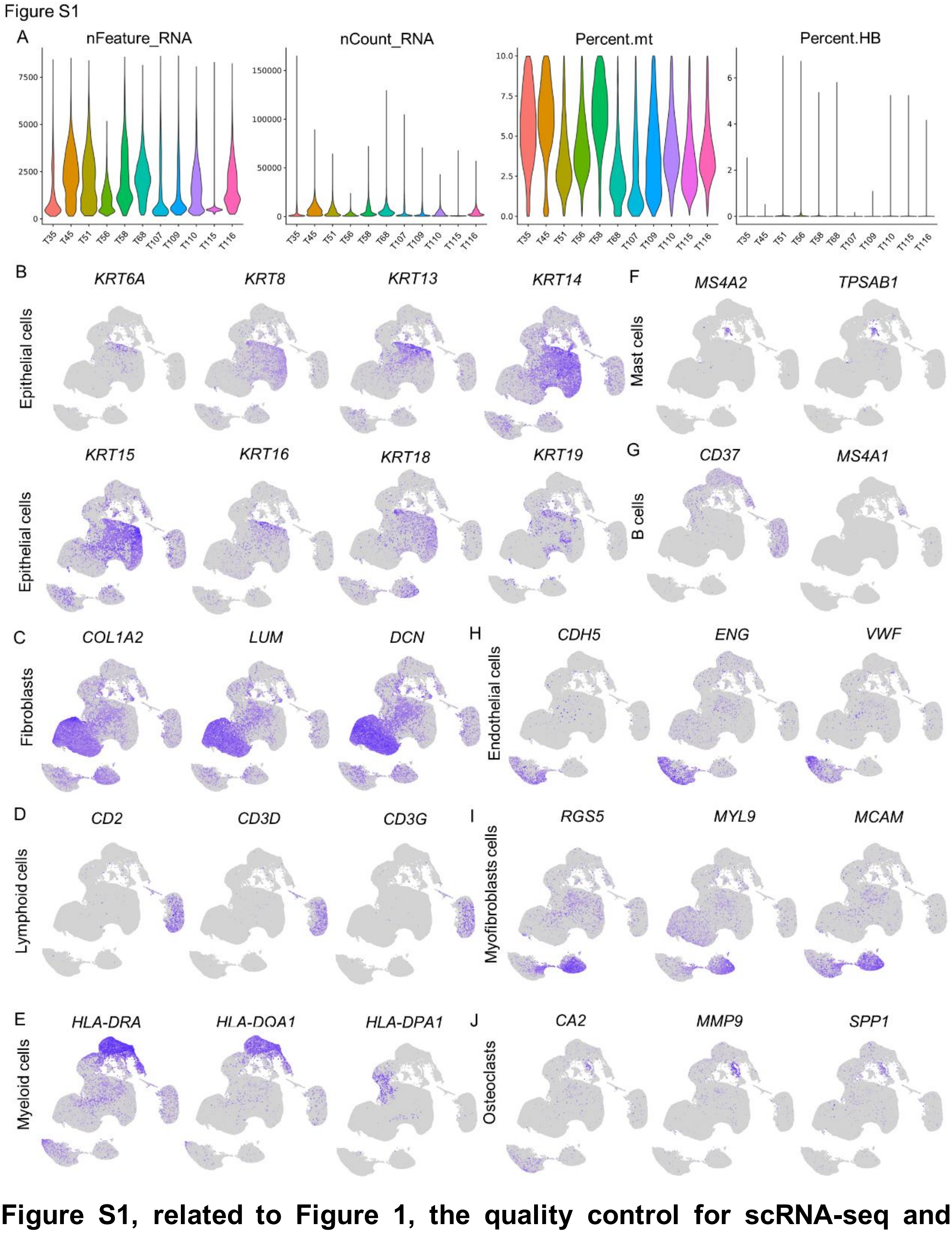


**Figure S1, related to Figure 1, the quality control for scRNA-seq and UMAP plot displaying the gene expression in different clusters.**

(**A**) Violin plot showing the number of genes (nFeature), number of UMI (nCount), percent of mitochondrial (percent.mt), and percent of hemoglobin (percent.HB) of each cell in each sample.

(**B-J**) UMAP plot displaying the expression of marker genes in the identified cell populations.

Figure S2

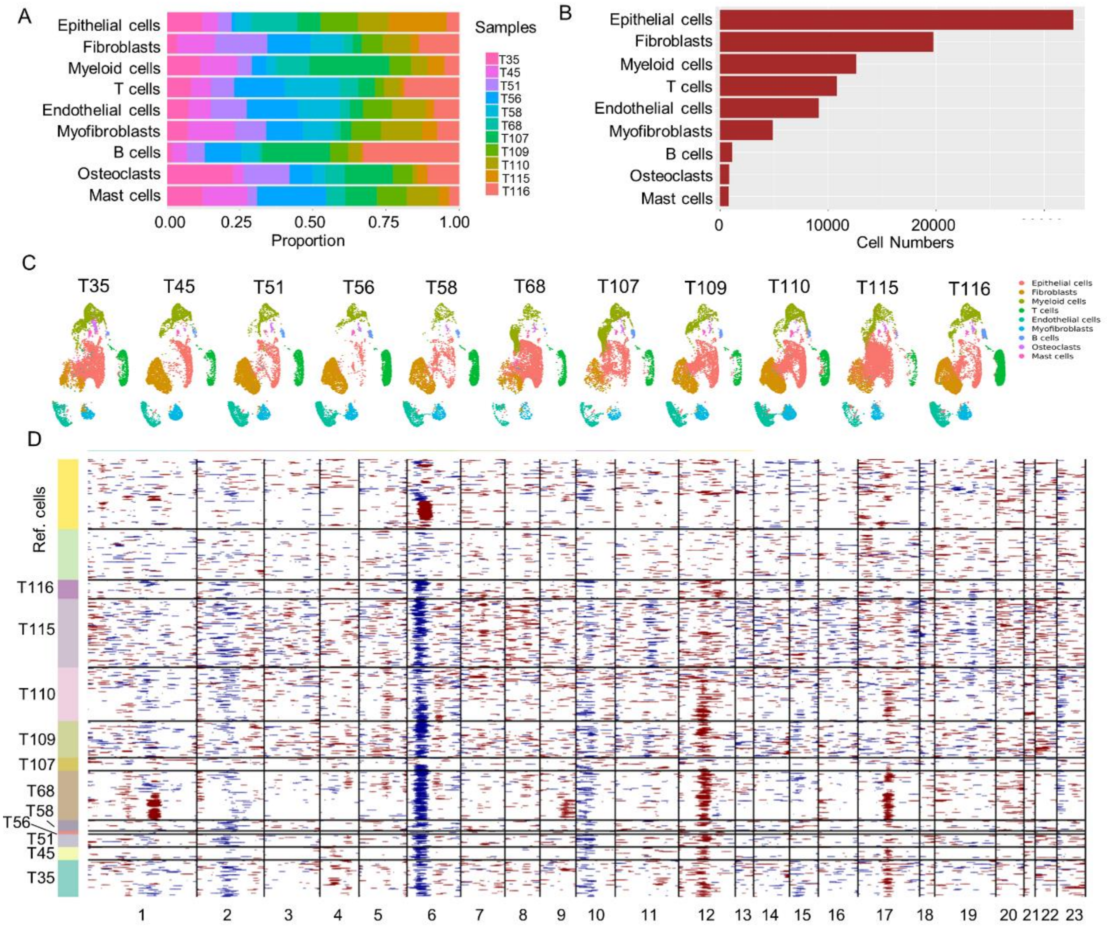


**Figure S2, related to Figure 1, the distribution of different cell types and inferCNV analysis.**

(**A**) Percentage of each tumor from patients in different cell types.

(**B**) Bar plot showing cell numbers in each cluster.

(**C**) UMAP plot distributing different clusters in each patient.

(**D**) Chromosomal heatmap showing large-scale CNVs in all ameloblastoma epithelial cells compared with reference cells (myeloid cells and T cells).

Figure S3

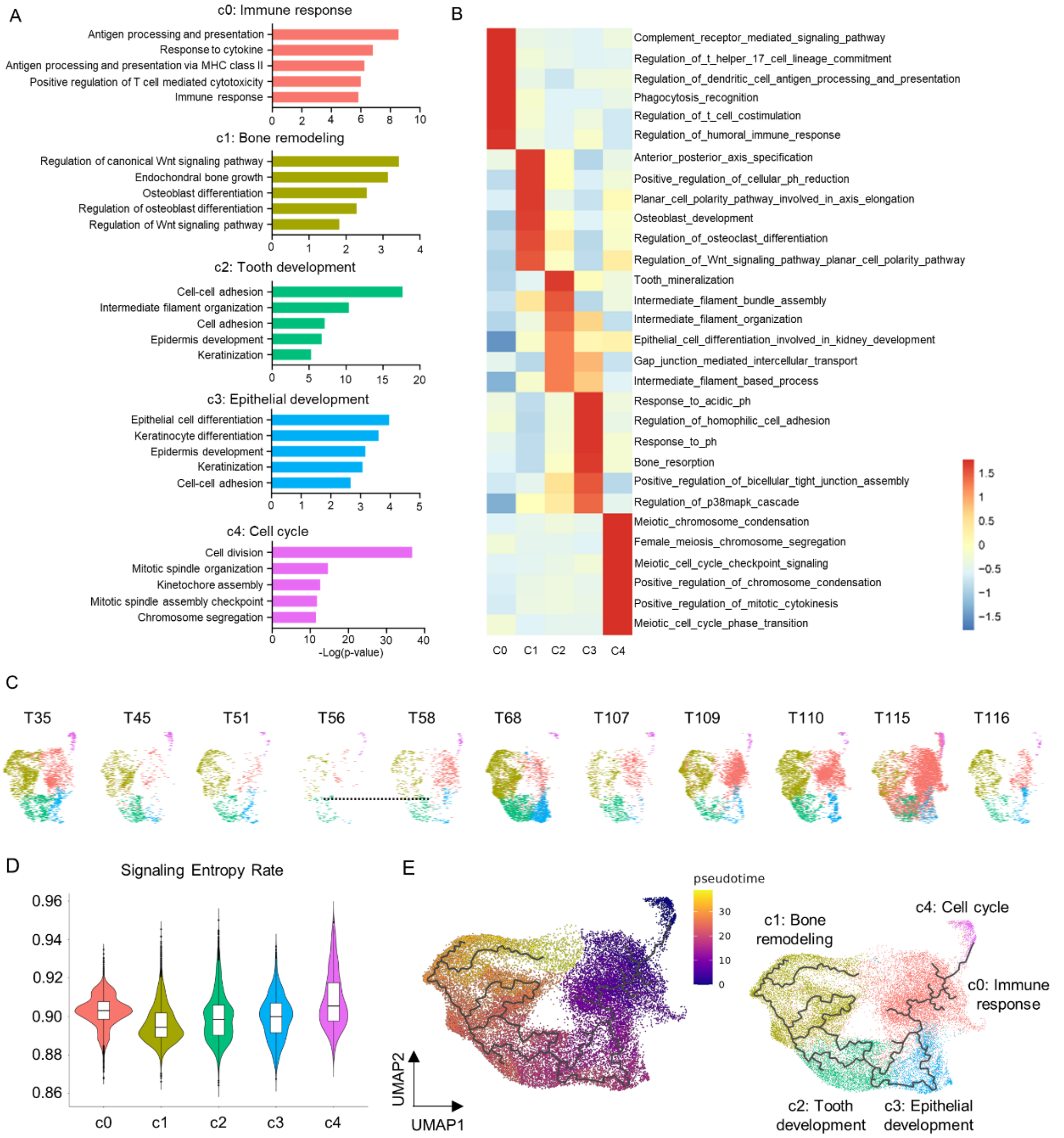


**Figure S3, related to Figure 2, GO, GSVA, signal entropy rate and trajectory analysis for different epithelial clusters.**

(**A**) Bar plots displaying the biological process from GO datasets for 5 epithelial clusters. The highly expressed genes in each cluster used for GO analysis.

(**B**) Heatmap showing differences in pathway activity of biological process between 5 epithelial clusters by GSVA. The scores of pathways are normalized.

(**C**) UMAP plot distributing different epithelial clusters.

(**D**) Violin plot displaying the signaling entropy rate of each cluster.

(**E**) Pseudotime evolution trajectory of epithelial cells inferred by monocle 3 analysis.

Figure S4

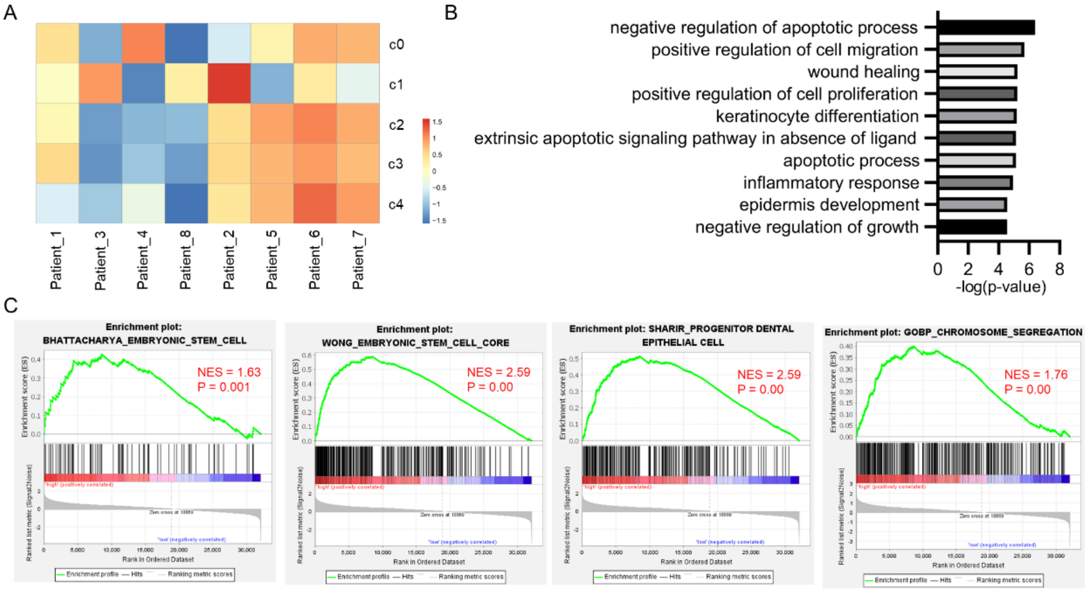


**Figure S4, related to Figure 5, high CC scores are correlated with cell cycle and stemness in ameloblastoma patient samples.**

(**A**) GSVA analysis showing the score of each epithelial cluster enriched in AM samples. The gene expression matrix of AM samples (GSE132472) was downloaded from GEO datasets.

(**B**) Bar plots showing the top 10 biological process from GO datasets for high CC score group. The highly expressed genes in high CC score group used for GO analysis (log2fold change > 1, p-value < 0.05).

(**C**) GSEA displaying that the stemness and cell cycle-associated signature were significantly enriched in high CC score group compared to low CC score group.

Figure S5

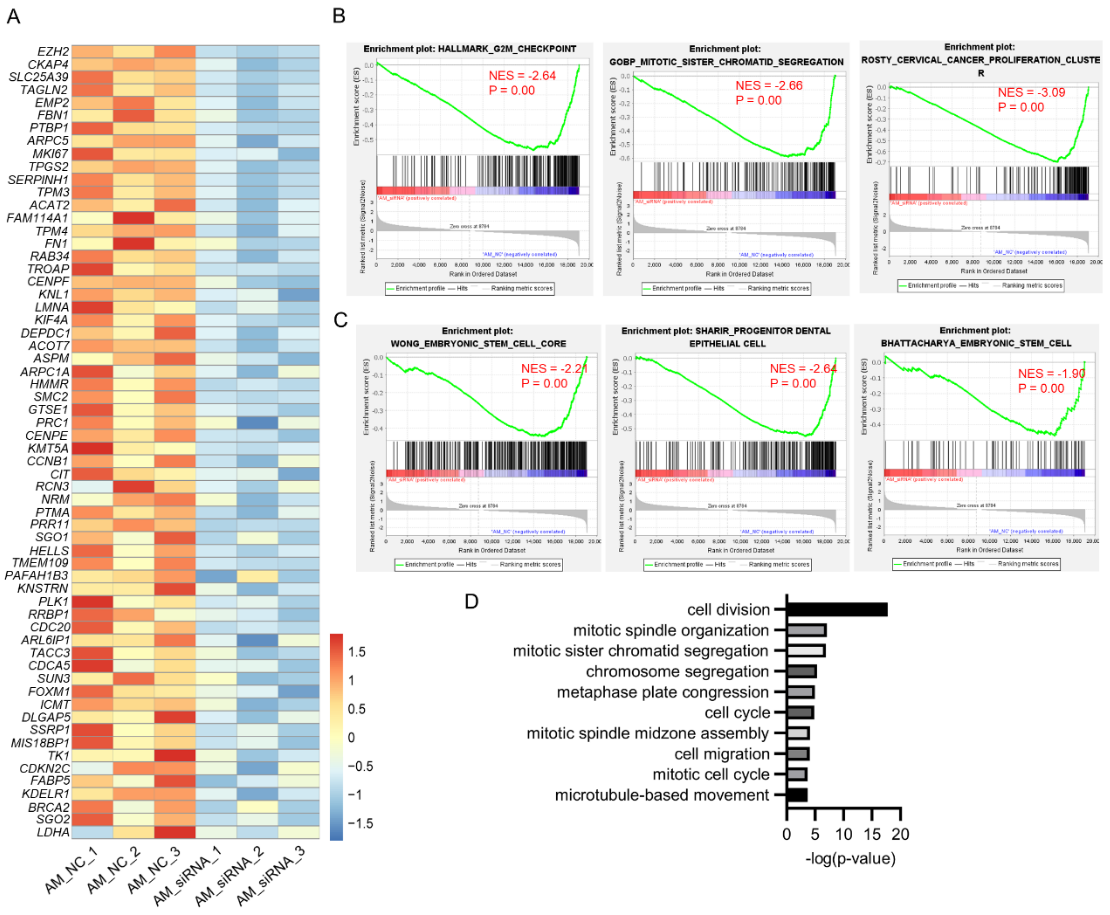


**Figure S5, related with Figure 6, EZH2 regulates proliferation and stemness in ameloblastoma cells.**

(**A**) Heatmap showing the expression of selected genes in AM cells transfected with control siRNAs and EZH2 siRNAs by RNA-seq.

(**B**) GSEA displaying that the cell cycle and proliferation-associated signature were significantly suppressed in AM cells transfected with EZH2 siRNA.

(**C**) GSEA displaying that the stemness-associated signature was significantly suppressed in AM cells transfected with EZH2 siRNA.

(**D**) Bar plots showing the top 10 biological process pathways from GO analysis for AM cells transfected with EZH2 siRNA. The DEGs were used for GO analysis (log2fold change > 0.5, p-value < 0.05).

Figure S6

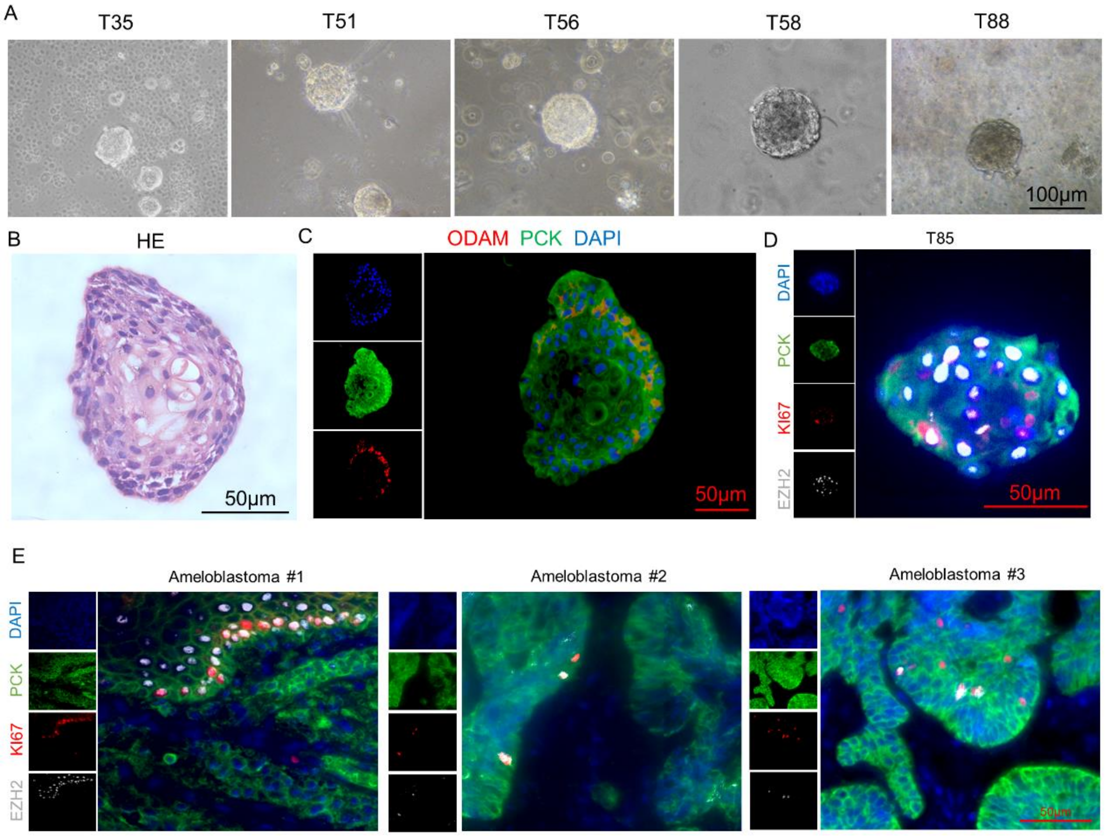


**Figure S6, related to Figure 7 and Figure 8, identification of the ameloblastoma patient-derived organoids and the co-expression of EZH2 and KI67 in ameloblastoma.**

(**A**) Representative images showing the organoids derived from different patients with ameloblastoma; scale bar, 100 μm.

(**B**) Hematoxylin and eosin (H&E) staining of organoid sections; scale bar, 50 μm.

(**C**) Representative immunostaining images showing the expression of PCK and ODAM in ameloblastoma organoids; scale bar, 50 μm.

(**D**) Representative images of multiplex immunohistochemistry showing the expression of PCK, KI67 and EZH2 in ameloblastoma organoids. Scale bar, 50 μm.

(**E**) Representative images of Multiplex immunohistochemistry showing the expression of PCK, KI67 and EZH2 in ameloblastoma samples; scale bar, 50 μm.
